# Complementary alternative health care in Israel and the western world

**DOI:** 10.1186/2045-4015-1-8

**Published:** 2012-02-20

**Authors:** Jeffrey Borkan

**Affiliations:** 1 Department of Family Medicine, MHRI-111 Brewster Street, Pawtucket, Rhode Island, USA

## Abstract

Complementary alternative health care presents fascinating challenges and opportunities for health policy and research - from issues of definition to training and licensing, to insurance reimbursement and evidence regarding clinical efficacy and outcomes. Complementary alternative health care is utilized by a substantial proportion of the population both now and likely in the future and requires serious health policy consideration.

This is a commentary on http://www.ijhpr.org/content/1/1/7/.

## Commentary

Complementary alternative health care (CAHC) presents fascinating challenges and opportunities for health policy and research - from issues of definition to training and licensing, to insurance reimbursement and evidence regarding clinical efficacy and outcomes. The article by Shuval and Averbuch [1] provides a remarkable overview of developments in this field in Israel, a country where such practices are both widespread and mainstream. As the authors note, over 1.7 million visits are estimated to take place annually in a country of less than 8 million people - and 65% of CAM services in Israel occur and are integrated into the public sector through the major health (sick) funds.

Clearly defining complementary alternative health care is the likely starting point for any discussion of this topic and, like many aspects of CAHC, it is filled with controversy. Although ***complementary and alternative health care ***is more comprehensive than the commonly used term ***complementary and alternative medicine ***(CAM), the latter is utilized in the article to refer to array the plethora of "non-conventional health practices" frequently used in Western societies. Even the question of what is CAM or if CAM even exists is a topic of debate. As noted by the *NIH National Center for Complementary and Alternative Medicine, "*Complementary and alternative medicine (CAM) is the term for medical products and practices that are not part of standard care." [2,3] This definition is one of exclusion - treatments that are not standard are thus considered ***alternative ***if used instead of standard ones or ***complementary ***if they are used in tandem with standard care. What constitutes "standard care" in any society is dynamic over time and varies widely from country to country based on everything from ever changing laws and regulations to payment mechanisms and even immigration patterns.

For example, treatments such as osteopathic or chiropractic manipulation in the United States have entered the mainstream over the past few decades, with numerous state laws, licensing of practitioners, and third party insurance payments. These acts have ensured that osteopathy and chiropractic have been virtually enshrined in the "standard care" category. Similarly, in parts of Europe, homeopathy has been included in standard care, something that has been rarely if ever broached in the U.S. Immigrant ethnic groups, especially those from less developed nations, often bring their traditional healing traditions with them to their host countries; however, strictly speaking such practices only exist the realm of "traditional medicine:" and enter the realm of CAM when Westerners from outside the ethnic group utilize them.

Other methods of definition have focused on the classification of the literally hundreds or thousands of health practices by modality or by theoretical roots. Others have skirted the issues of classification, focusing instead on efficacy of medical and therapeutic practices. The question here becomes not one of conventional or non-conventional, but rather, ***does the therapy in question work?***, i.e., does it have evidence of scientifically tested effectiveness. Shuval and Averbuch [1] also point out that the definition of bio-medicine versus CAM is in part defined by what is taught in medical schools. In Israel, as in many countries, the teaching of CAM never makes it beyond medical school elective options, if it enters the curriculum at all.

There are a host of other key issues regarding CAM that Shuval and Averbuch [1] cover in their article that should be of interest to the broad health policy and health care audience. One important issue is governmental regulation and control. As Shuval and Averbuch [1] note, despite the widespread use of CAM practices in Israel, there is relatively little governmental control and, to date, no formal jurisdictional regulation or licensing procedures have been established. Though often ascribed to the domination of bio-medicine in Israel, other important factors that have resulted in this regulatory chasm may be the sheer number and literal cacophony of treatment modalities, providers, and training standards. Having done research on CAM in Israel in the past and recently informally sampled CAM in two areas in Israel, I am impressed by the spectrum of practices that vary from common choices such as acupuncture to offshoots of esoteric movement therapies to spirit mediums. Regulating and controlling such a range of therapeutic modalities would be challenging, if not impossible.

The authors' conclusion will have to withstand the test of time: "CAM is here to stay and its use is likely to increase in Israel in future years." My sense is that this is likely true, though the form in which it manifests itself in the future may be starkly different than what we see today. As the authors suggest, this article should be "useful to health policy planners in other countries in which CAM provides increasing portions of health care", though Israeli health planners may themselves need to learn its lessons.

## Competing interests

The authors declare that they have no competing interests.

## Author's information

Jeffrey Borkan, MD, PhD is Professor and Chair of the Department of Family Medicine at the Alpert Medical School of Brown University. He is active in health policy and health policy research, now in the US (currently the Chair of the US Council for Academic Family Medicine - CAFM), and previously in Israel.
